# Expression of TrkC Receptors in the Developing Brain of the *Monodelphis* *opossum* and Its Effect on the Development of Cortical Cells

**DOI:** 10.1371/journal.pone.0074346

**Published:** 2013-09-03

**Authors:** Katarzyna Bartkowska, Monika Gajerska, Kris Turlejski, Rouzanna L. Djavadian

**Affiliations:** Department of Molecular and Cellular Neurobiology, Nencki Institute of Experimental Biology, Warsaw, Poland; Universitat Pompeu Fabra, Spain

## Abstract

In this study, we investigated the distribution, localization and several various functions of TrkC receptors during development of the *Monodelphis* *opossum* brain. Western blotting analysis showed that two different forms of the TrkC receptor, the full-length receptor and one of its truncated forms, are abundantly expressed in the opossum brain. The expression of TrkC receptors was barely detected in the brain of newborn opossums. At postnatal day (P) 3, the expression of full-length TrkC remained at low levels, while moderate expression of the TrkC truncated form was detected. The expression levels of both forms of this protein gradually increased throughout development, peaking at P35. We found that in different neocortical areas located both at the rostral and caudal regions of the cortex, up to 98% of BrdU-labeled cells forming cortical layers (II-VI) had prominently expressed TrkC. To assess which developmental processes of cortical cells are regulated by TrkC receptors, three different shRNAs were constructed. The shRNAs were individually tested in transfected cortical progenitor cells grown on culture plates for 2 days. The effects of the shRNA-TrkC constructs were similar: blockade of TrkC receptors decreased the number of Ki67-positive and apoptotic cells, and it did not change the number of TUJ-positive neurons *in vitro*. Thus, the lack of TrkC receptors in cultured progenitor cells provided insight on the potential role of these receptors in the regulation of proliferation and cell survival but not in the differentiation of cortical cells.

## Introduction

The highly diversified mammalian infraclass Marsupialia split from the infraclass Eutheria at least 125 million years ago, and since then, they have evolved in parallel [1], independently developing similar ecological specializations. The marsupial family Didelphidae, which lives only in America, is considered to be the core family of Marsupials. Many Didelphid species, including the gray short-tailed opossum *Monodelphis* *domestica* show anatomical features that are similar to those of early marsupials and eutherians [2].

The general pattern of brain development in marsupials is similar to that in eutherians. However, marsupial newborns have extremely immature brains due to a short gestation period. Thus, neurogenesis in the diencephalon and telencephalon generally occurs after birth during postnatal life [3–8]. The sequence of neocortical development in the gray short-tailed opossum ( *Monodelphis* *domestica* ) is similar to that observed in other mammals [9,10]. The cortical plate first appears at P3, and it gradually expands and thickens until P16. The neocortical layers form in an inside-out manner, which indicates that the first layers to develop are the deep layers VI and V, and subsequently, the consecutive upper layers are formed with the exception of the oldest cells in layer I, which are a remnant of the cortical plate [2,9,10]. However, the role of neurotrophin receptors in the mechanisms of marsupial brain development remains unknown. Numerous studies in the eutherian species have shown that the expression of neurotrophins starts at a very early stage of brain development [11,12]. Interestingly, different neurotrophins and their receptors are selectively expressed at various stages of development, and they control crucial developmental processes such as cell proliferation and differentiation, survival, synaptogenesis and neuronal morphology [13–17].

Neurotrophin-3 (NT-3) and its preferred tropomyosin-receptor-kinase C (TrkC) receptor are widely expressed in eutherian brains [18]. Six different isoforms of the TrkC receptor have been identified. Isoforms of TrkC, which are generated by alternative splicing of the intracellular domain (truncations or insertions), specifically respond to NT-3. Two TrkC isoforms (TrkC-T1 and TrkC-T2) are generated by a truncation of the intracellular tyrosine kinase domain, and three isoforms (TrkC-14, TrkC-25 and TrkC-39) are produced by the insertion of amino acids of different (14, 25 or 39 amino acids, respectively) lengths [19-21]. Functionally, distinct TrkC isoforms may modulate signal transduction pathways by forming heterodimers with full-length receptors or by competitive ligand binding. Activation of truncated TrkC receptors can inhibit full-length TrkC receptors and function as dominant-negative receptors [22,23]. However, data on neurotrophins and their receptors in marsupials are limited, particularly on TrkC receptors and their isoforms in the opossum brain.

In the current study, we first investigated the expression of TrkC (full-length and truncated) in the *Monodelphis* *opossum* brain at different developmental time points. Next, the inhibitory effect of TrkC on the development of cortical progenitor cells cultured from opossum brains at P1 and at P7 was investigated using genetic constructs.

## Materials and Methods

### Animals

Opossums born in the Nencki Institute colony were used in this study. The animals were housed under a 14/10 h light/dark cycle and had ad libitum access to food and water. All efforts were taken to minimize the number of animals used and the amount of stress placed on them. Experimental procedures complied with the Polish Law on Experimentation on Animals, which implements the European Council Directive of 24 November 1986 (86/609/EEC) as well as with the NIH Guide for the Care and Use of Laboratory Animals. The experiments were approved and controlled by First Warsaw Local Ethics Committee for Animal Experimentation (Permit Number: 766/2007). All animals were sacrificed with pentobarbital anesthesia, and all efforts were made to minimize suffering.

### Western blotting analyses

Opossums at P1, P3, P7, P12, P20, P35, P60 and adults (over one year old) were sacrificed by decapitation. Each group contained a minimum of three animals. The opossum brains were quickly dissected and frozen at -70°C. Isolated brain regions were homogenized in lyses buffer containing protease inhibitors and detergents and then subjected to Western blotting analysis. Protein samples (30 µg/lane) were loaded on a 9% SDS-PAGE and electroblotted onto a nitrocellulose membrane for 2 h at 380 mA, at 4°C. The blots were blocked in 5% skimmed milk powder dissolved in Tris-buffered saline with 0.2% Tween 20 for 2 h at room temperature and then incubated overnight at 4°C with one of the following primary antibodies: rabbit anti-TrkC (C44H5) from Cell Signaling (1:1200), rabbit anti-TrkC (798) and rabbit anti-TrkC (H-300) (both 1:200, Santa Cruz Biotechnology) or mouse anti-GAPDH protein (1:10000, Chemicon). After several washes, the blots were incubated with secondary goat anti-rabbit antibodies conjugated with horseradish peroxidase (1:7000, BioRad Laboratories) or goat anti-mouse antibodies conjugated with horseradish peroxidase (1:10000, Chemicon) for 2 h at room temperature. Detection was performed using an enhanced chemiluminescence reagent (ECL Kit, Amersham Bioscience) followed by X-ray film exposure.

To determine the specificity of the TrkC antibody, a blocking peptide was used. Before performing the staining, the antibody was incubated with a peptide that corresponded to the antibody-recognizing epitope. The peptide was incubated with an equal volume of antibody (1:1) or 10- and 50-fold more peptide was added to the antibody solution. In all cases, no signal was visible on the Western blot.

### BrdU injections and perfusion

To determine the fate of newly generated cells in the brain, we used opossum pups at P1, P3, P5, P7, P9, P12, P14 and P17. The animals received subcutaneous injections of BrdU at a single dose of 20 mg/kg. All injected animals were anesthetized with pentobarbital (100 mg/kg) and perfused transcardially with saline (0.9% NaCl) followed by 4% paraformaldehyde in 0.1 M phosphate buffer, pH 7.4 at the age of 3 months.

### Histology and immunocytochemistry

After perfusion, the brains were removed from the skulls, postfixed in a fixative solution and soaked in 30% sucrose prior to sectioning. The brains were cut into 40-µm sections on a cryostat. Ten series of the sections were collected.

For BrdU immunohistochemistry, free-floating sections were incubated for 2 hours at 60°C in 50% formamide dissolved in saline sodium citrate buffer and then rinsed with saline sodium citrate buffer. The sections were subsequently incubated in 2 M HCl at 37°C for 30 min and then rinsed with a 0.1 M solution of boric acid (pH 8.5) for 10 min. Next, the sections were rinsed three times in Tris buffer (0.1 M, pH 7.5), transferred to 1% H_2_O_2_ for 30 min and then rinsed with Tris buffer containing 0.1% Triton X-100 and 0.05% bovine serum albumin. For the next hour, the sections were incubated in the Tris buffer containing 10% normal goat serum, and incubated overnight in the primary antibody solution (mouse monoclonal anti-BrdU antibody from Roche, diluted 1:1000 in Tris with 0.1% Triton X-100 and 0.05% bovine serum albumin). This antibody (clone BMC 9318) was prepared by immunizing mice with a conjugate of BrdU and bovine serum albumin, and it should not cross-react with any endogenous cellular components. After several washes, the sections were incubated for 1 hour in secondary anti-mouse antibody diluted in Tris buffer (1:200), washed again and incubated for 1 hour in an extravidin-peroxidase complex diluted in Tris buffer (1:200). Both the secondary antibody and extravidin complex were purchased from Sigma. The sections were rinsed three times in Tris buffer and then reacted with diaminobenzidine enhanced with nickel salts (DAB Substrate Kit, Vector). The sections were mounted on to slides, dried and coverslipped with DePeX (Serva).

Immunofluorescent double-labeling procedures were performed using the following combined antibodies: anti-BrdU and anti-TrkC, anti-TrkC and anti-neuronal nuclei protein (NeuN), anti-glial fibrillary acidic protein (GFAP) or anti-oligodendrocyte lineage gene 2 (Olig2) antibodies. The deeper cortical layer marker transducing-like enhancer of split 4 (Tle4) combined with anti-TrkC was also used. The following primary antibodies were used: rabbit anti-TrkC (1:500, Cell Signaling), mouse anti-NeuN (1:200, Millipore), mouse anti-Tle4 (1:100, Santa Cruz), mouse anti-GFAP (1:500, Sigma), mouse anti-Olig2 (1:100, Millipore), and rat anti-BrdU (1:300, Santa Cruz). Secondary antibodies included anti-rabbit Alexa Fluor 568 (1:500, Molecular Probes), anti-rabbit Alexa Fluor 647 (1:500, Molecular Probes), anti-mouse Alexa Fluor 488 (1:500, Molecular Probes), anti-rat (1:200, Jackson Immunoresearch) and streptavidin-DTAF (1:300 Jackson Immunoresearch).

To identify the different cortical layers, selected sections were counterstained with DAPI (1:5000, Sigma).

### Cultures, transfections and immunostaining

Cortical precursor cells obtained from P1 or P7 opossum neocortices were dissociated in ice-cold phosphate buffered saline (PBS) and transferred into Neurobasal medium (Invitrogen) supplemented with 2 mM GlutaMAX, 2% B27, 1% penicillin – streptomycin (all from Invitrogen), 0.6% glucose (BioWet) and 40 ng/ml FGF2 (Sigma). The tissue was mechanically triturated using a plastic pipette and plated into four-well chamber slides (Nunc) that were precoated with 2% laminin (Sigma) and 1% poly-D-lysine (Sigma) at a density of 130,000 cells/well. Three to four hours after plating, 1 µg DNA and 3 µl X-tremeGENE 9 DNA Transfection Reagent (Roche) was mixed with 100 µl of OptiMEM (Invitrogen) and incubated at room temperature for 30 minutes prior to addition into each well. We performed the transfections using a control plasmid pGPU6/GFP/Neo-shRNA (negative control) and three TrkC shRNA plasmids, which targeted three different regions on the TrkC opossum mRNA sequence. The sequence for shRNA-TrkC1 was 5’-CACCGCAGTAAGACAGAGATCAATTTCAAGAGAATTGATCTCTGTCTTACTGCTTTTTTG-3’, for shRNA-TrkC2 was 5’-CACCGCATCAACATCACGGACATCTTTCAAGAGAAGATGTCCGTGATGTTGATGCTTTTTTG-3’, for shRNA-TrkC3 was 5’-CACCGGAGCTCTATACTGGACTTCATTCAAGAGATGAAGTCCAGTATAGAGCTCCTTTTTTG-3’, and for the control shRNA was 5’- CACCGTTCTCCGAACGTGTCACGTCAAGAGATTACGTGACACGTTCGGAGAATTTTTTG3’.

The control shRNA was mismatched to known human and mouse genes (EZBiolab). Each of the plasmids encoded a green fluorescent protein (GFP), which was used as a transfection marker.

After 48 hours in culture, the cells were fixed for 10 min using 4% paraformaldehyde, permeabilized with 0.2% NP-40 (Roche) in PBS and blocked with a buffer containing 6% normal goat serum and 0.5% bovine serum albumin (Sigma) for 1–2 hr at room temperature. The cells were then incubated with the following primary antibodies: monoclonal anti-Ki67 (1:150, DACO), polyclonal anti-cleaved caspase 3 (1:750, Cell Signaling) or polyclonal anti-TUJ (1:750, Covance) and monoclonal or polyclonal anti-GFP (1:750, both from Millipore) at 4°C overnight. After several washes in PBS, the cells were incubated with secondary antibodies at room temperature for 1 hr. The secondary antibodies included anti-rabbit Alexa Fluor 568 (1:750, Molecular Probes) and anti-mouse Alexa Fluor 488 (1:750, Molecular Probes). The cells were then washed with PBS, counterstained with DAPI (1:5000, Sigma) for 10 min, and mounted with Mounting Medium (Sigma).

### Data analysis

The optical density (OD) of the full-length TrkC, truncated TrkC and GAPDH protein bands was measured using the Gene Tools Software (Syngene) with a densitometric measurement feature. The OD value of each band was established by subtracting the OD level of the background. One-way ANOVA was used to compare the means between different ages or brain structures.

Immunostained sections were imaged and analyzed using Neurolucida. Colocalization of double and triple fluorescent labels was determined using a confocal laser microscope (Zeiss). The percentage of double-labeled cells of at least 200 TrkC-positive cells in the cerebral cortex of each animal was quantified and analyzed. Next, the number of TrkC and BrdU, BrdU and NeuN, TrkC and NeuN, TrkC and GFAP, TrkC and Olig2 colocalized cells was quantified.

For the double-labeled cultured cells, between 100 and 400 transfected cells (depending on the efficiency of the culture) per condition per experiment were quantified and analyzed. Statistics were performed using one-way ANOVA, and the error bars indicate the S.E.M.

## Results

### Expression of TrkC receptors in the brain during development

Western blotting analysis detected changes in the expression of TrkC receptors throughout the postnatal stages of brain development in the opossum. The expression of TrkC receptors was examined from postnatal (P) day 1 (P1; day of birth) and continued until P3, P7, P12, P20, P35, P60 or P400 (13 months). The last selected developmental point corresponded to a middle-aged adult opossum.

**Figure 1:**
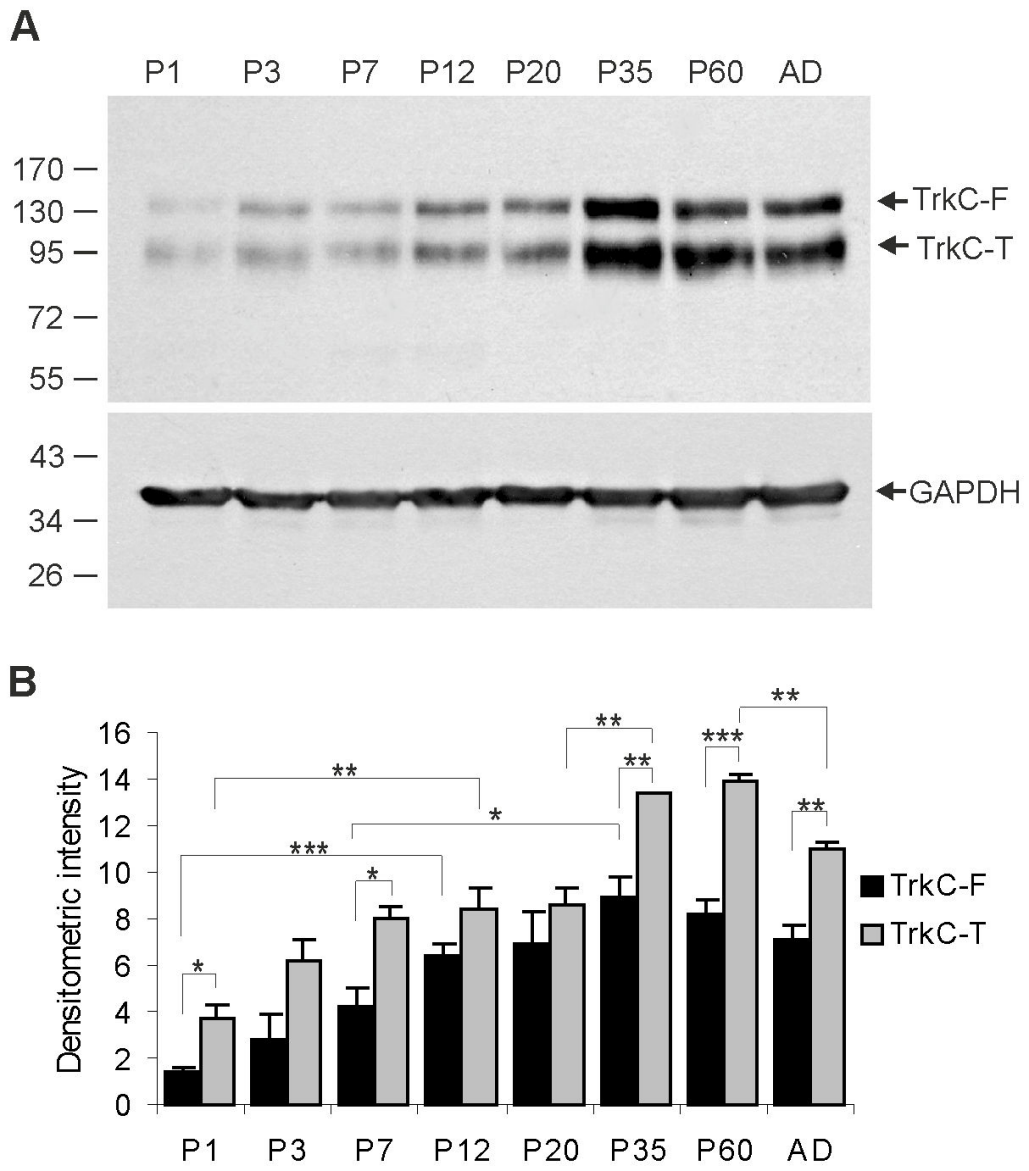
Expression of TrkC receptors in the developing opossum brain. A - Western blotting analysis was performed in opossum brain homogenates at different ages (P1, P3, P7, P12, P20, P35, P60 and adult). GAPDH was used as a loading control. The arrows indicate the positions of the full-length (TrkC-F) and truncated (TrkC-T) TrkC receptors. B - graph illustrates the mean amount of TrkC protein from three different animals at each age for both full-length (black bars) and truncated forms (gray bars) of TrkC receptors. Error bars indicate ± S.E.M. Note that the highest level of TrkC expression was observed at P35 and P60. * indicates P<0.05; ** indicate P<0.01; *** indicate P<0.001 for all figures.

To detect TrkC protein in the opossum brain, three different antibodies were used. However, two of the antibodies were not specific to the opossum brain. Western blotting analysis was performed using whole brain lysates. Although we detected several labeled bands on the immunoblots using TrkC (H-300) and TrkC (798) antibodies, none of these proteins recognized the 145-kDa neurotrophic TrkC receptor protein. However, the TrkC (C44H5) antibody revealed the expression of one of the truncated forms and the full-length form of the TrkC receptor in whole brain lysates (Figure 1 A). The full-length form of the TrkC receptor (TrkC-F, fully glycosylated protein) exhibits a molecular mass of approx. 145 kDa. Full-length TrkC was slightly expressed in newborn animals and its expression significantly increased by the end of the first week of life. Its levels continued to increase until day 20 and remained unchanged thereafter (Figure 1 B). One-way ANOVA showed that there was a statistically significant difference between groups (F_7,23_ = 9.89 p<0.001). Holm-Sidak multiple comparisons also showed that there were significant differences between P1 and P12; P1 and P20, P35, P60 and adult; P3 and P35; and P7 and P35 animal groups. The same antibody detected another band with a molecular weight of 95 kDa, which corresponded to one of the truncated forms of the TrkC (TrkC-T) receptor. Its low-level expression was detected in newborn opossum brains and then gradually increased until P35. The highest expression level of this truncated protein was observed at P35, the stage when the opossum starts to open its eyes. The truncated protein expression decreased slightly in later development and remained at a stable level in adult animals (Figure 1 A, B). Statistically significant differences were also observed between groups (F_7,23_ = 32.4 p<0.001).

In all of the examined developmental periods, the expression of truncated TrkC receptors (95 kDa protein) was higher compared to the full-length form of TrkC (Figure 1 B).

**Figure 2:**
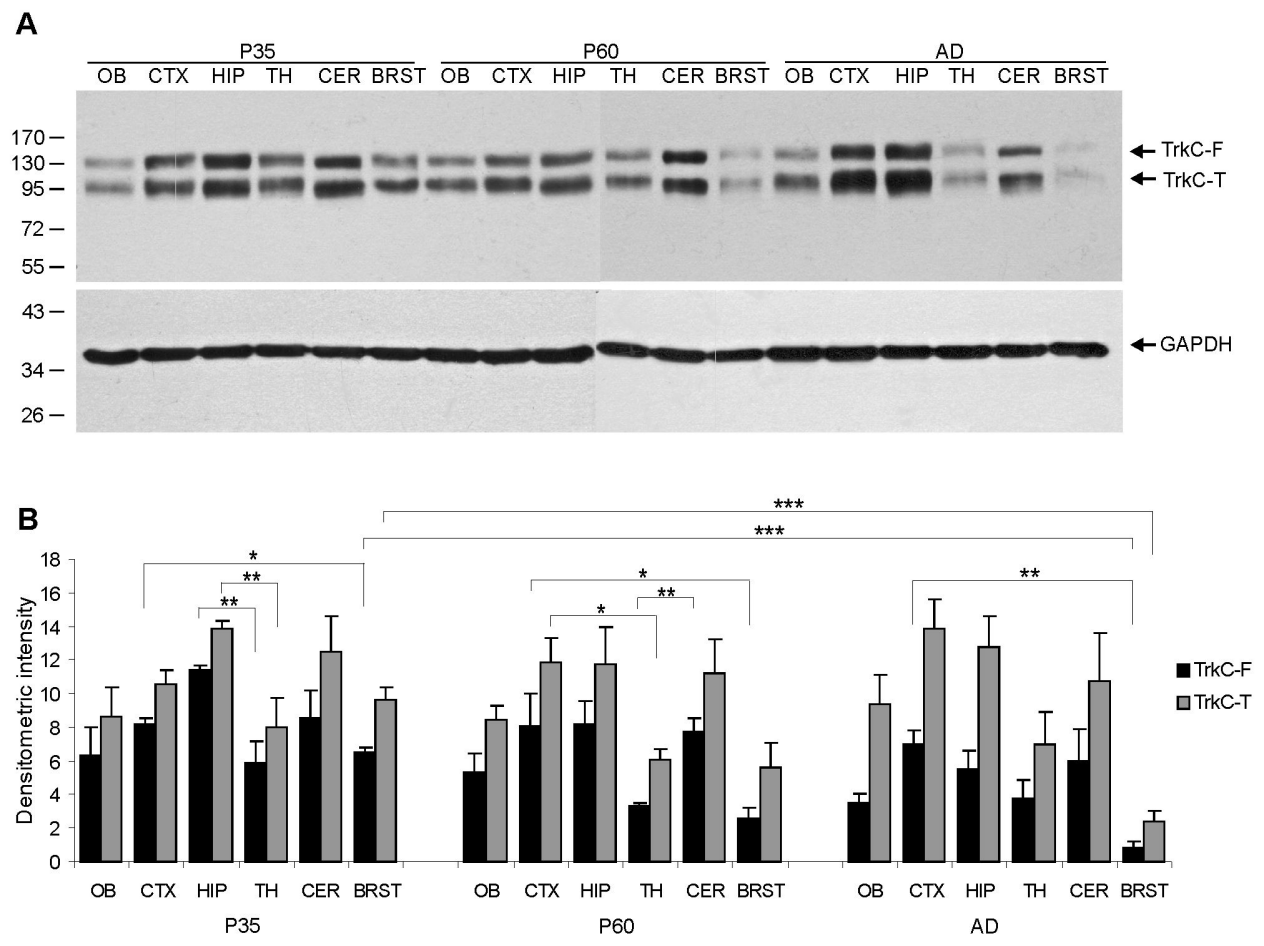
Expression of the full-length and truncated TrkC receptors in different brain structures in the opossum. A - Western blotting analysis was performed in 6 different regions of the brains at P35, P60 and adults: OB –olfactory bulb, CTX –cerebral cortex, HIP –hippocampus, TH –thalamus, CER –cerebellum, and BRST – brainstem structures. GAPDH was used as a loading control. The arrows indicate the positions of the full-length (TrkC-F) and truncated (TrkC-T) TrkC receptors. B – graphs showing the amount of both forms of TrkC receptors in the brain structures. Error bars indicate ± S.E.M.

To study the distribution of TrkC receptors various brain regions were isolated using dissection. This method was performed over three stages of development: P35, which corresponded to the time of eye opening, P60, a period when the opossums are weaned, and P400, when opossums are fully adult. At early ages, the opossum brains were too small to collect some of the brain regions. Strong expression of both full-length and truncated forms of TrkC was observed in the hippocampus, cerebellum and cerebral cortex. However, slightly lower expression was found in the thalamus and olfactory bulb at P60 and in adult opossums (Figure 2 A). Both full-length and truncated TrkC were detected at very low levels in the brainstem structures at P60 and in adult animals (Figure 2 B). We observed that the level of TrkC expression was 6-7 times higher in the adult cerebral cortex compared to other brainstem structures (p<0.006). Interestingly, in all of the examined structures, expression of the truncated form of the TrkC receptor was higher compared to the expression of its full-length form, specifically in the adult cerebral cortex (p<0.02).

### Expression and localization of TrkC in the cerebral cortex

**Figure 3:**
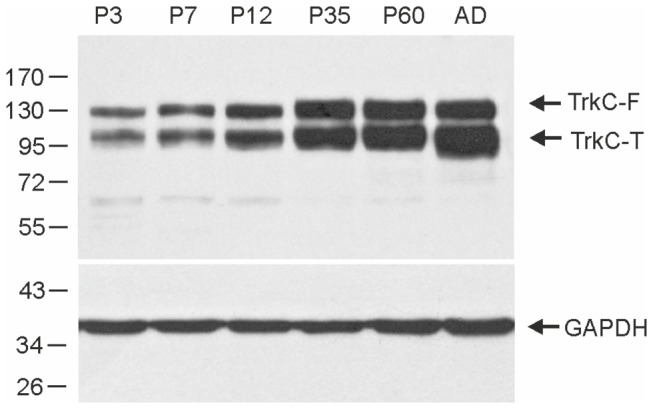
Expression of TrkC in the developing cerebral cortex of the opossum. A – Western blotting analysis was performed at P3, P7, P12, P35, P60 and adult animals. GAPDH was used as a loading control. The arrows indicate the positions of the full-length (TrkC-F) and truncated (TrkC-T) TrkC receptors.

Newborn opossums weighed 108.4 ± 20.6 mg, where their average brain weight was 4.8 ± 1.3 mg and the average weight of the cerebral cortex was 1.3 ± 0.5 mg. However, the homogenized tissue obtained from one opossum cerebral cortex was not sufficient for Western blotting. Thus, cortical tissue lysates were prepared from the pooled cortices of 3-5 opossums at P3, P7 and P12. For older ages, lysates containing the left and right hemispheres from each animal were used. We found that TrkC expression was low at P3, when cortical layer formation begins (Figure 3 A). However, it increases at P7 and P12 (Figure 3 A), a stage when intensive histogenesis occurs, forming cortical layers (the results are presented below).

**Figure 4:**
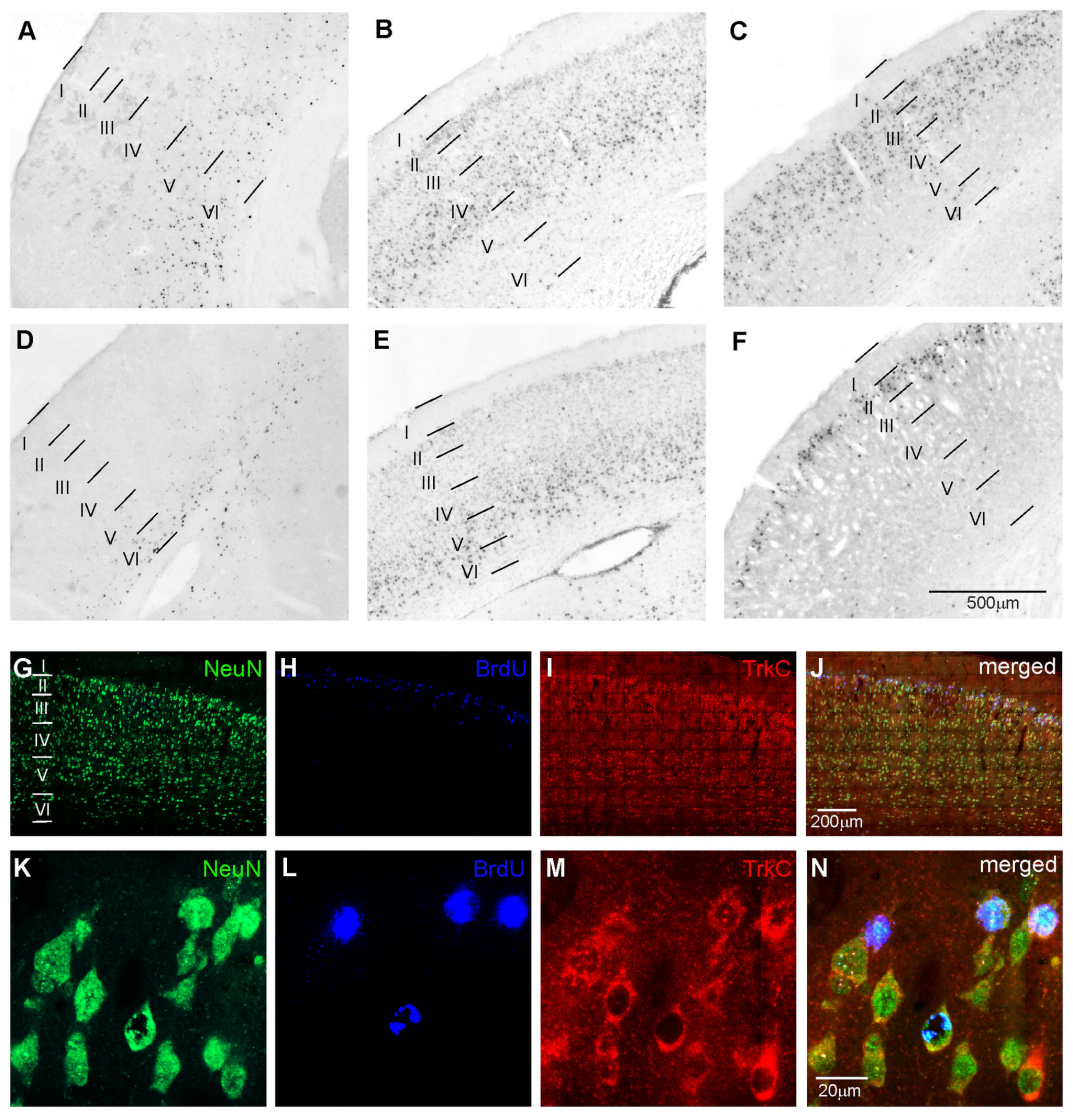
Expression of TrkC in newly generated BrdU-labeled cells, which form layers in the cerebral cortex. A-F - Location of the BrdU-labeled cell nuclei on coronal brain sections in 3-month-old opossums injected with BrdU on days P3 (A, D), P7 (B, E) and P12 (C, F). Two sections per brain are illustrated. A-C – Sections were obtained from the rostral part of the opossum brains. D-F - Sections were obtained from the caudal part of the opossum brain. Animals were injected with BrdU at 3 (D), 7 (E) and 12 (F) days after birth. G-L – confocal images of double staining for BrdU and TrkC coexpression in the same neurons. A coronal section from the caudal part of the opossum brain that was injected 12 days after birth and perfused at 3 months.

To examine the birthdates of cells in different cortical layers, the opossums were injected with BrdU at various developmental time points, and then perfused at the age of 3 months. The long survival period after BrdU injections determine the final location of the labeled cells in the brain at specific developmental time points. We found that at P3, a clear rostroventral-to-caudodorsal gradient of BrdU-immunoreactivity was present in the cortical layers (Figure 4 A, D). At P3, many BrdU-labeled nuclei were found in the infragranular layers VI and V (Figure 4 A) and in layer IV of the rostroventral region of the neocortex, where the frontal, cingulate and somatosensory areas are located. However, the immunoreactive BrdU cells were restricted only to layer VI and partially to layer V of the caudodorsal neocortex, which largely corresponded to visual area 17 (Figure 4 D). The emergence of layers IV and III of the rostroventral cortex (Figure 4 B) and layers V and IV of the caudodorsal cortex occurred at P7 (Figure 4 E). The last cortical layer of the rostrodorsal cortex was established at P12 (Figure 4 F), while generation of cortical neurons in the caudodorsal part of the cortex ended at P17 (data not shown). In addition, the vast majority of BrdU-labeled cells expressed TrkC and NeuN (Figure 4 G–L).

**Figure 5:**
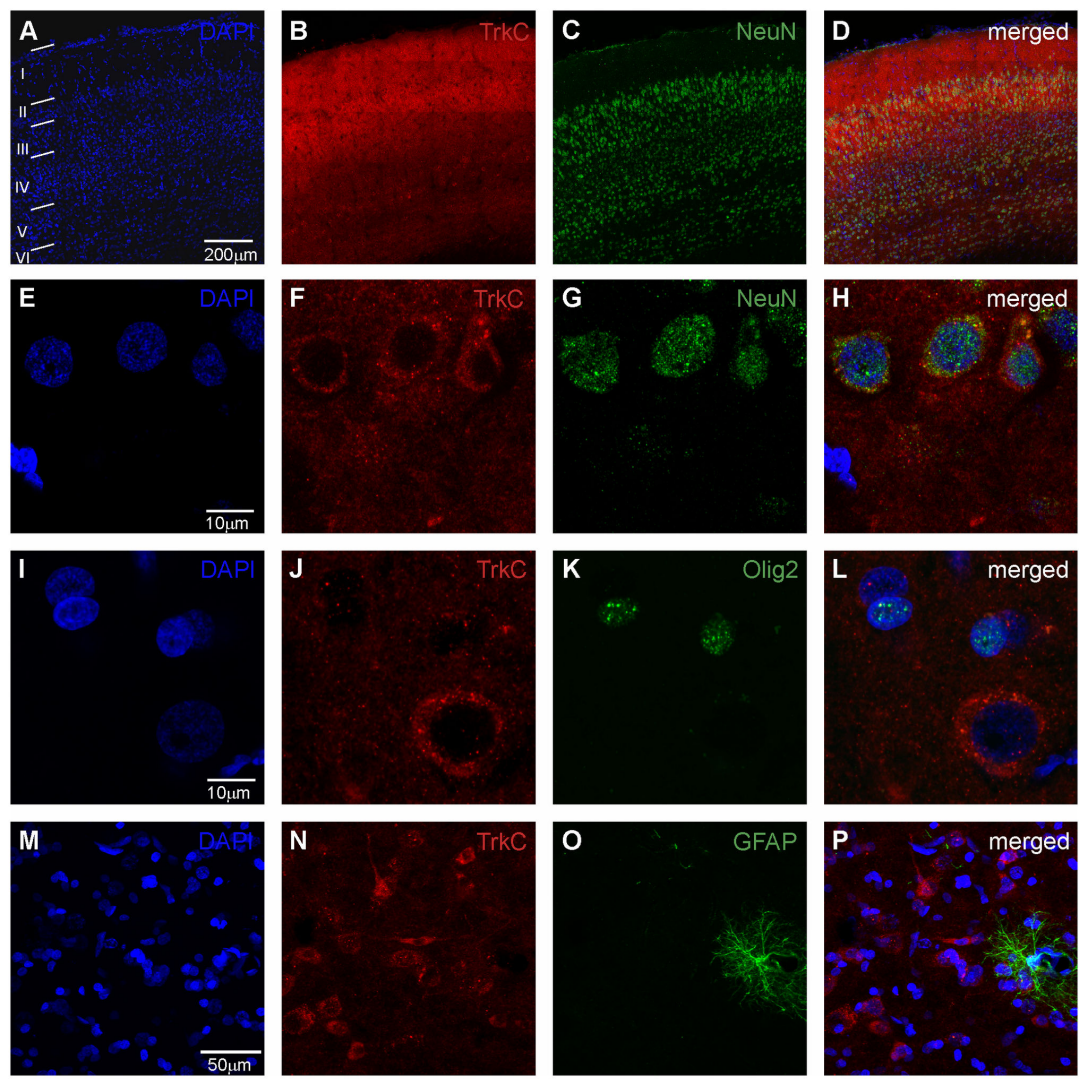
Phenotype of cortical cells expressing TrkC receptors. Low-magnification (A–D) and high-magnification (E–P) confocal images showing triple immunofluorescence staining of cortical sections. Immunostaining was performed with DAPI (blue) to label the cell nucleus, TrkC receptors with an antibody against TrkC (red) and neurons (G, H) with NeuN antibody (green), or oligodendrocytes (K, L) with Olig2 (green), or astrocytes (O, P) using an antibody against GFAP (green).

To determine the phenotype of cortical cells expressing TrkC receptors, triple immunofluorescence staining was performed and high-magnification confocal images were obtained (Figure 5). We analyzed TrkC-expressing cells in the opossum cerebral cortex that were injected with BrdU at P3, P7 and P12. A total of 250-270 TrkC-expressing cells per animal were quantified in two specific regions (the anterior and caudal part of the cerebral cortex). In the neocortex, nearly all (98%) of the TrkC-labeled cells prominently expressed NeuN and demonstrated a neuronal phenotype (Figure 5 E–H). Moreover, glial cells also expressed TrkC receptors. In addition, the number of double-labeled cells was very low in the cerebral cortex. Approximately 1% of cells (for example, 2 cells from 250 TrkC cells in one case) were co-labeled with anti-GFAP, a marker for astrocytes, TrkC receptors (Figure 5 M–P). Cells labeled using the oligodendrocyte marker Olig2 were often located very close to the soma of the TrkC neurons but were never colocalized in the same cell (Figure 5 I–L).

**Figure 6:**
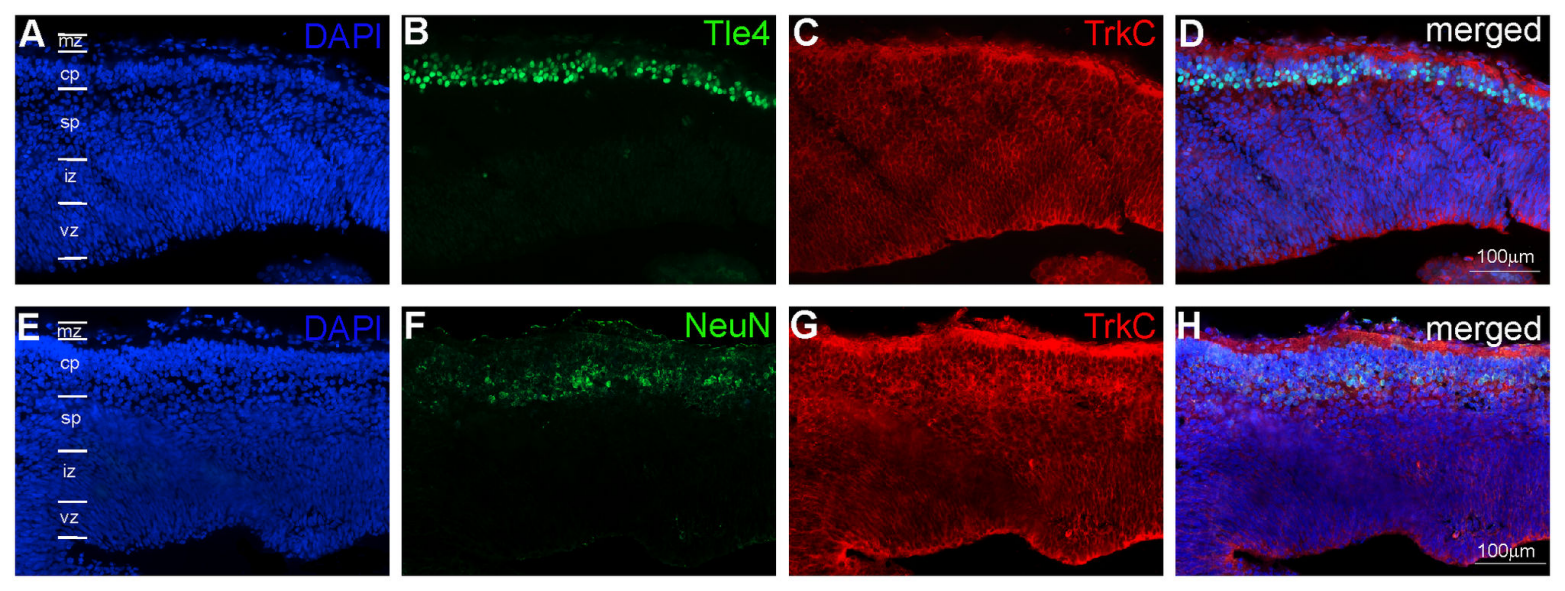
Coexpression of TrkC with the markers Tle4 and NeuN in neocortical cells at P7. A-D – triple labeling for DAPI (A), Tle4 (B) and TrkC (C) or DAPI (E), NeuN (F) and TrkC (G), and all three labels were merged in D and H, respectively. Analysis of the images using a confocal microscope showed that in both cases, nearly all the Tle4 or NeuN cells expressed TrkC. In A and E, the cortical layers are marked. Scales in D and H refer to all images.

To characterize whether the cells forming cortical layers have a neuronal phenotype, we stained developing opossum brains for Tle4 and TrkC or NeuN. Nearly all Tle4-labeled cells coexpressed TrkC and NeuN (Figure 6).

### TrkC signaling in cortical progenitor cells

The role of TrkC receptors in mechanisms of cortical cell development was examined using genetic constructs (shRNA) that silenced gene expression and reduced TrkC receptor expression in cells. Progenitor cells isolated from the cerebral cortex at P1 or P7 were transfected with pGPU6/GFPshRNA plasmid and then cultured in vitro for 2 days. We selected two stages of development at P1, when the cortical layers had not yet formed, and at P7, when half of the cortical layers had already formed. In both cases, the progenitor cells were transfected with either a control plasmid (control) or with three different plasmids that blocked endogenous TrkC receptor expression (shRNA-TrkC1, shRNA-TrkC2, shRNA-TrkC3).

**Figure 7:**
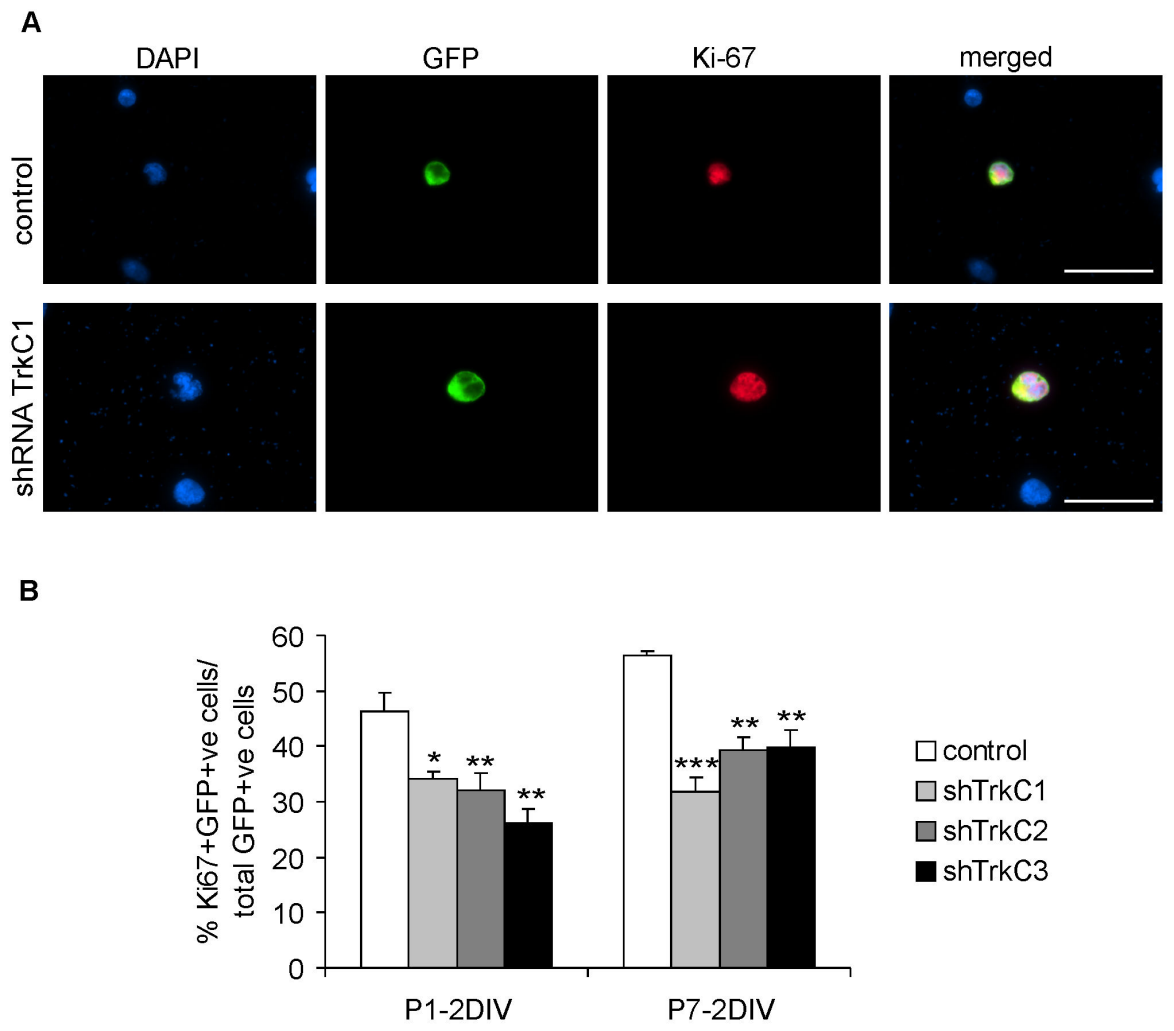
Effect of TrkC receptor downregulation on cortical cell proliferation. A –triple-labeling (DAPI/GFP/Ki-67) of cortical progenitor cells cultured from P1 opossums in control conditions (transfected with control shRNA) and cells transfected with shRNA-TrkC1. Scale bars: 30 µm. B – graphs showing the percentage of dividing cortical cells in control (transfected with control shRNA) and transfected (shRNA-TrkC1, shRNA-TrkC2 and shRNA-TrkC3) cultures obtained from opossums at P1 (P1-2DIV) and P7 (P7-2DIV).

The effect of TrkC receptor activity on the proliferation of cortical progenitors was examined using an antibody against the Ki-67 protein, which is present only in the nuclei of dividing cells (Figure 7 A). In control conditions, the percentage of dividing cells in cultures isolated at P1 was approximately 50% (Figure 7 B). Blockade of the endogenous TrkC receptor using shRNA-TrkC1, shRNA-TrkC2 or shRNA-TrkC3 decreased the rate of proliferation by approximately 30% (Figure 7 B). In cell cultures obtained from older animals (P7), the inhibition of TrkC receptors also decreased the rate of cell proliferation (Figure 7 B). One-way ANOVA showed a significant difference between groups (F_3,11_=10.24 P<0.004). All pairwise comparisons using the Holm-Sidak method showed significant differences in the number of Ki-67-labeled cells between cultures transfected with the control plasmid and those transfected with either the TrkC1, TrkC2 or TrkC3 plasmids.

**Figure 8:**
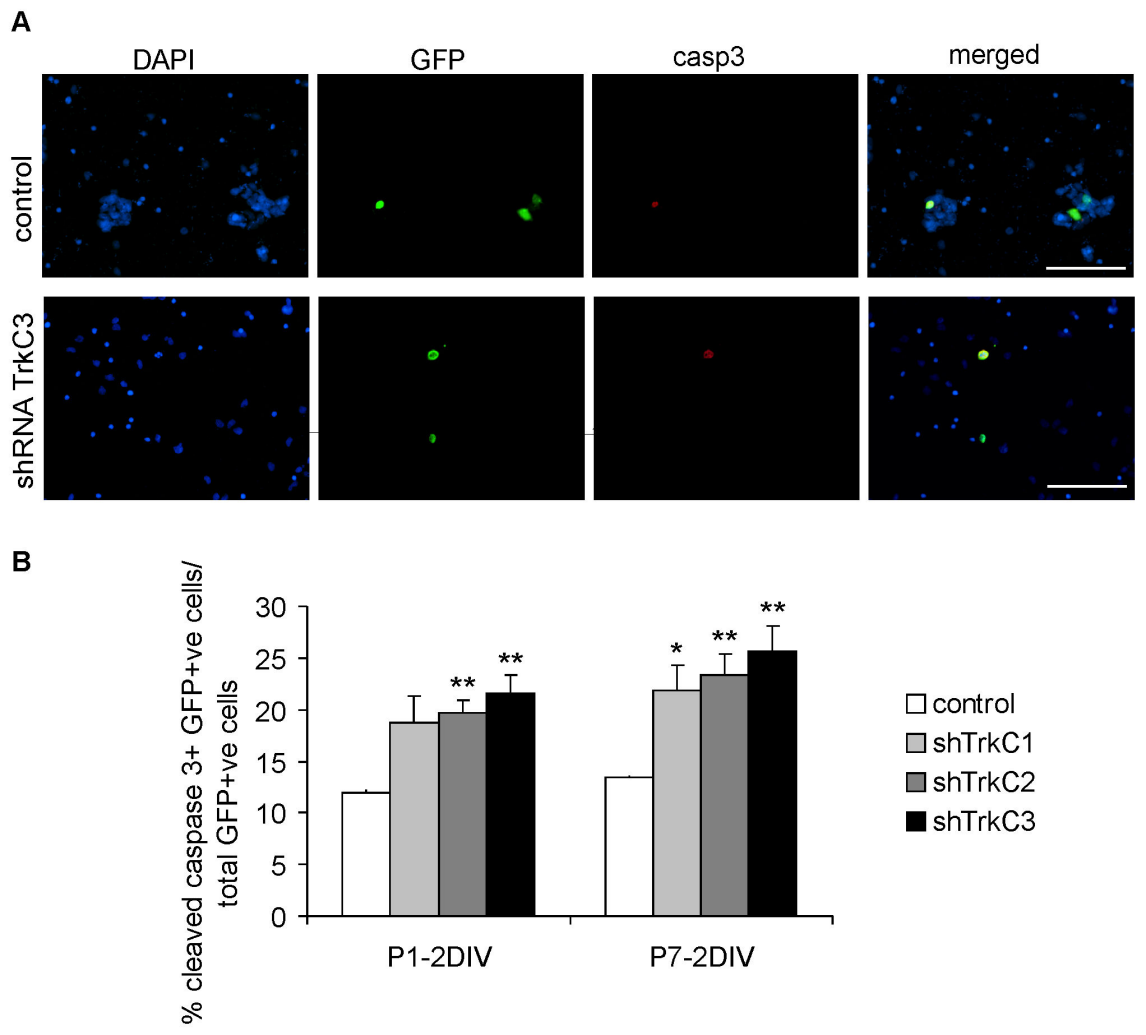
Effects of TrkC receptor downregulation on cortical cells apoptosis. A – triple-labeling (DAPI/GFP/casp3) of cortical progenitor cells cultured from P1 opossums in control conditions (transfected with control shRNA) and cells transfected with shRNA-TrkC3. Scale bars: 100 µm. B – graphs showing the percentage of apoptotic cells (transfected with control shRNA, shRNA-TrkC1, shRNA-TrkC2 and shRNA-TrkC3) in cultures obtained from opossums at P1 (P1-2DIV) and P7 (P7-2DIV).

Cell survival was determined using an antibody recognizing cleaved caspase-3, which is expressed in dying cells undergoing apoptotic death (Figure 8). In control conditions, approximately 10% of GFP-positive cells (i.e., transfected with the control plasmid) were apoptotic, while transfections using the shRNA-TrkC plasmid increased the number of apoptotic cells (Figure 8 B). The highest rate of apoptosis was found in cell cultures transfected with shRNA-TrkC3 (25%) and shRNA-TrkC2 (20%) constructs. Similarly, in cell cultures obtained from P7 animals, the percentage of dying GFP-positive cells in control conditions was decreased (approximately 10%), while inhibition of TrkC receptor activity increased the number of dying cells by two-fold (Figure 8 B).

**Figure 9:**
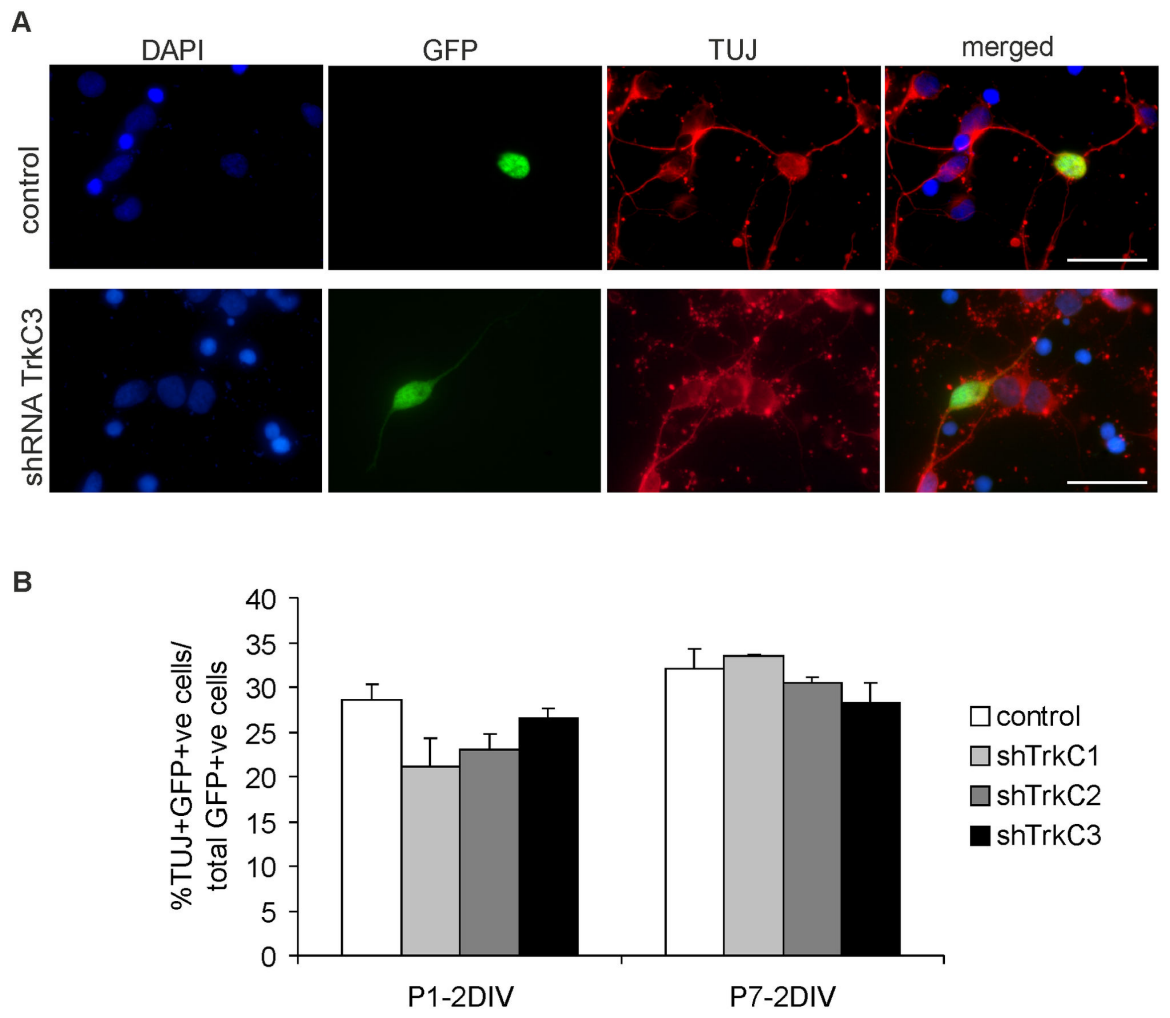
Effects of reduced activity of TrkC on the neuronal differentiation of cortical cells. A - triple-labeling (DAPI/GFP/TUJ) of control (transfected with the control plasmid) and shRNA-TrkC3 transfected cortical progenitor cells cultured from opossums at age P7. Scale bars: 25 µm. B – graphs showing the percentage of neurons (cells stained with neuron-specific β3-tubulin) in cultured cells transfected with control shRNA, shRNA-TrkC1, shRNA-TrkC2 and shRNA-TrkC3 obtained from opossums at P1 (P1-2DIV) and P7 (P7-2DIV).

To examine the effect of reduced TrkC receptor activity on the differentiation of isolated cortical cells, immunofluorescent staining using a neuron-specific marker, β3-tubulin (TUJ), was performed (Figure 9). The number of TUJ-positive neurons was approximately 30% in control cell cultures in P1 opossums, whereas this number decreased for cells transfected with shRNA-TrkC1 (Figure 9 B). In contrast, there was no effect on cell differentiation in cell cultures derived from P7 opossums (Figure 9 B).

## Discussion

We investigated the expression of full-length and truncated TrkC receptors during opossum brain development. TrkC was expressed at early stages of brain development, and its expression gradually increased with the growth of the brain, peaking at P35. However, slightly reduced but strong expression was observed in the adult opossum brain. TrkC receptors were heterogeneously distributed throughout various brain structures. In the cerebral cortex, the level of TrkC expression progressively increased throughout development. Nearly all BrdU-labeled neurons generated on specific days during laminar cortical development also expressed TrkC. During early development, TrkC receptors regulated several mechanisms that control cell development. In particular, they were involved in proliferation and apoptosis but not in cell differentiation.

To study the distribution of TrkC receptors in the opossum brain, we tested several antibodies generated based on previous literature of the *Monodelphis* *opossum* genome DNA sequences. In the protein databank, we found original peptide sequences for opossum TrkC receptors (Ensembl Genome Browser at www.ensembl.org). We compared the sequences of commercially available antibodies and selected sequences with a minimum of 80-95% homology to the opossum sequences, one of which proved to be specific. Our Western blotting analyses showed two specific protein bands for TrkC receptors, one of which corresponded to the full-length form of TrkC. Previous studies on placental mammals have shown that the *trk*C locus is complex and may encode at least nine different isoforms [21,24–27]. Truncated forms of TrkC are conserved in rat, chicken and mouse [28–31]. TrkC receptors are activated by selective binding of its ligand NT-3. Moreover, the amino acid sequences for NT-3 have been shown to be similar among placental animals, monotremes and marsupials [32]. Furthermore, Forooghian et al. [27] reported that truncated isoforms of TrkC in the cat were similar to those found in rat [28] and human [33].

The Trk family of receptors is evolutionarily conserved. However, studies on similar receptors in invertebrates have been performed on only one invertebrate, the mollusk *Lymnaea*. In this species, the gene for the Ltrk receptor conserves the same order of exons and introns as the mammalian *trk* gene [34,35]. Although we did not study the opossum *trk* gene, our data demonstrated the expression of TrkC receptors in this marsupial species, which suggests that TrkC receptors are conserved during evolution.

We found that truncated TrkC receptors exhibit higher expression levels compared to the full-length form of TrkC during opossum brain development. However, a different pattern of TrkC expression was observed in the brains of eutherian mammals. During embryonic development, the expression levels of full-length TrkC receptors were abundant in eutherian brains, including humans, while the expression levels of truncated TrkC receptors were expressed postnatally [29,31,36]. In humans, the levels of the full-length and truncated forms of TrkC were comparable until 17 years of age, after which the truncated form is predominately expressed [36]. However, the function of TrkC receptor isoforms is still poorly understood. Mice overexpressing truncated TrkC receptors die within the first postnatal week [37]. Moreover, transgenic mice lacking all TrkC isoforms demonstrate a neuronal deficiency [38–40].

*Monodelphis* opossums are born at earlier stages of development compared to rodents [41]. In newborn opossums, brainstem structures and the cervical segment of the spinal cord are developmentally more advanced. Their activity enables the newborn opossum pup to control their breathing, generate some movements and to suck milk [42]. This stage of development in the opossum brain is comparable to the mouse embryo brain at day 12 [43]. At birth, TrkC receptors are present in the opossum brain but at a very low level. In mice, the onset of TrkC expression corresponds to the formation of the neural tube at embryonic day 8.5 [11]. At 11.5-day mouse embryo TrkC transcripts were observed in the central nervous system in nearly all cortical regions, including the telencephalon. During later embryogenesis, TrkC is differentially expressed in several brain structures, where its expression remains broad throughout postnatal brain development in rodents [11,12]. On the basis of these data, it has been suggested that TrkC receptors may play diverse functions in early brain development. Although we have not investigated TrkC expression in the opossum embryo brain, it appears likely that the expression of TrkC in the mouse embryo brain begins earlier (embryonic day 8.5) compared to the opossum, where its expression begins at P1.

In the opossum brain, the expression of TrkC receptors increased during the first postnatal week and was associated with robust neurogenesis in the midbrain and forebrain. The increase in TrkC protein in the cerebral cortex from P3 to P12 coincided with the development of the cortical laminae, which were generated according to an inside-out pattern in a clear rostroventral-to-caudodorsal gradient. In eutherians, developmental cortical neurogenesis occurs in utero [44,45], and in species of a size comparable to the opossum, it lasts for a few days. In mice, cortical neurogenesis begins at E12 and is finished by E18 [46,47]. Thus, the neurons in neocortical layers VI-II are generated over a 6-day period [48]. In the opossum, this process lasts 10 days in the rostroventral region and 14 days in the caudodorsal region of the neocortex. Because of the slow developmental pace, the rostroventral-to-caudodorsal gradient of development in the opossum cerebral cortex is very clear, resulting in the development of rostral and caudal regions of the cerebral cortex at different times.

The highest levels of TrkC protein were detected at P35, an age when opossums begin to open their eyes. This is a period of maximal plasticity in the nervous system, when erroneous or unnecessary axon collaterals are pruned, remaining connections develop and the majority of synapses are formed [49]. Some features of visual and somatosensory cortical representations, such as the ocular dominance columns of the primary visual cortex (V1) and separation of the representation of different body parts in the primary somatosensory cortex (S1) emerge from the processes that occur during this time [50,51].

This developmental stage is also characterized by the generation of oligodendrocytes. In eutherians, gliogenesis follows neurogenesis. The same sequence has also been observed in the opossum [10]. We hypothesized that TrkC receptors might regulate the number of oligodendrocytes because a high level of TrkC receptor expression has been associated with the origin of oligodendrocytes. This suggestion was supported by data obtained from knockout mice revealing that the absence of TrkC signaling in mice resulted in the loss of Ia afferent projections to the spinal cord [38]. Moreover, TrkC knockout mice have a reduced number of myelinated axons, indicating the potential function of TrkC in the generation of oligodendocytes [52]. However, in the opossum, immunostaining using an antibody against Olig2 showed that several oligodendrocytes were located in close proximity to neurons, although none of the oligodendrocytes colocalized with TrkC. Thus, there was no direct effect of TrkC receptors on the origin of the oligodendrocytes.

Expression analyses of TrkC receptors in the opossum cortex revealed that TrkC protein levels increased when neurogenesis occurred and remained high when cell proliferation ceases. Thus, we postulated that TrkC, similar to eutherian mammals, might play various functions in the developing opossum brain [53–55]. To test this, disruption of TrkC signaling was performed in cultured cortical progenitors derived from P1 and P7 opossums. At P1, none of the cortical layers had yet formed, whereas at P7, three lower cortical layers (IV, V and VI) had already developed [10]. Our results showed that TrkC receptors can regulate cell survival. TrkC receptor interaction with its ligand results in the transduction of cell survival signals, mostly via the Ras-Raf-MEK-ERK and PI3K-Akt pathways [55,56]. Barnabe-Heider and Miller [57] reported that the TrkC signal could regulate cultured progenitor cell survival only through the PI3-kinase pathway in mice. Similar results were obtained from embryonic stem cells [58].

Recently, it has been shown that TrkC receptors are dependence receptors [59–61]. They transduce positive signals in the presence of the NT-3 ligand. However, they are also active in the absence of NT-3, and this activity triggers apoptosis. Thus, it is possible that a high level of TrkC receptors in opossum brains at P35 controls programmed cell death, where the receptors respond as dependence receptors. Thus, further investigations are required to confirm this hypothesis.

The number of differentiated neurons in cell cultures obtained from P7 opossums was not altered by the downregulation of TrkC receptors, while a slight reduction in neuronal differentiation occurred if the progenitor cells were isolated at P1. Thus, in contrast to mouse, inhibition of TrkC receptors in the opossum had no effect on neuronal differentiation processes [62]. However, our data were consistent with the results of other studies demonstrating that TrkC signaling is essential for the regulation of neuronal proliferation and cell survival [57,63].

## Conclusions

Taken together, the expression pattern of TrkC receptors in the opossum showed many similarities and some differences from that observed in rodents. Unlike in rodents, the truncated form of TrkC receptors dominated over the full-length form of TrkC receptors for nearly all developmental periods.

Evaluation of the specific differences in TrkC localization and distribution between eutherians and marsupials provided new opportunities for the understanding of some mechanisms of brain development. The sequences and mechanisms of brain development in these animals were very similar, while the differences were small.
